# Identification of Two GDSL-Type Esterase/Lipase Genes Related to Tissue-Specific Lipolysis in *Dendrobium catenatum* by Multi-Omics Analysis

**DOI:** 10.3390/life12101563

**Published:** 2022-10-09

**Authors:** Xinqiao Zhan, Yichun Qian, Bizeng Mao

**Affiliations:** 1 Institute of Biopharmaceuticals, Taizhou University, Taizhou 318000, China; 2 Institute of Biotechnology, Zhejiang University, Hangzhou 310000, China; 3 Ministry of Agriculture Key Lab of Molecular Biology of Crop Pathogens and Insects, Hangzhou 310000, China; 4 Key Laboratory of Biology of Crop Pathogens and Insects of Zhejiang Province, Hangzhou 310000, China

**Keywords:** *Dendrobium catenatum*, lipase, multi-omics, expression pattern, gene family

## Abstract

*Dendrobium catenatum* is an important herb and widely cultivated in China. GDSL-Type Esterase/Lipase proteins (GELPs) are widely distributed in plants and play crucial roles in stress responses, plant growth, and development. However, no identification or functional analysis of GELPs was reported in *D. catenatum*. This study identifies 52 GELPs in *D. catenatum* genome, which is classified into four groups by phylogenetic analysis. Four conservative blocks (Ser-Gly-Asn-His) are found in most GELP domains. Transcriptome analysis reveals the expression profiles of *GELPs* in different organs and flowering phases. Co-expression analysis of the transcriptome and lipidome identifies a GELP gene, *Dca016600*, that positively correlates with 23 lipids. The purified Dca016600 protein shows the optimum pH is active from 8.0 to 8.5, and the optimum temperature is active from 30 °C to 40 °C. The kinetic study provides V_max_ (233.43 μmol·min^−1^·mg^−1^) and K_m_ (1.49 mM) for substrate *p*-nitrophenyl palmitate (*p*-NPP). Integrated analysis of the transcriptome and proteome identifies a GELP gene, *Dca005399*, which is specially induced by freezing. Interestingly, *Dca005399* shows high expression in symbiotic germination seeds and sepals. This study provides new insights into the function of *D. catenatum* GELPs in plant development and stress tolerance.

## 1. Introduction

GDSL-Type Esterase/Lipase proteins (GELPs) are a variety of hydrolytic enzymes with broad substrate specificity and regiospecificity, with thioesterase, protease, arylesterase, and lysophospholipase activity [1]. The conserved GDSL motif contains four invariant important catalytic residues Ser, Gly, Asn, and His, which are also named SGNH hydrolases [2]. GELPs contain many members and widely exist in plants, such as more than 100 members in rice [3], 105 members in *Arabidopsis* [4], and 194 members in soybean [2]. GELPs have been suggested to play crucial roles in plant development and metabolism. In rice, most GELPs are highly expressed in germinating seeds and are responsible for lipid homeostasis [5]. GELPs also modulate phytohormone signaling in plant growth. A GDSL lipase gene (*LIP1*) in *Arabidopsis* is induced by GA and repressed by DELLA proteins, which mediates the enhanced germination potential [6]. GELPs are involved in auxin-induced processes of suberin polymerization and degradation in root development [7]. *MHZ11* encodes a GDSL-family lipase with acyl-hydrolyzing activity and is induced by ethylene. MHZ11 also acts with the ethylene receptor ETHYLENE RESPONSE SENSOR2 (OsERS2) and impairs CONSTITUTIVE TRIPLE RESPONSE2 (OsCTR2) phosphorylation for triggering ethylene signaling in rice [8]. The *DAD1* (*defective in anther dehiscence1*) gene belongs to the GELP family and encodes a particular phospholipase A1 (PLA1) that participates in jasmonic acid (JA) biosynthesis and linolenic acid metabolism. The defects of *DAD1* lead to anthers dehiscence, pollen maturation, and flower opening [9]. Moreover, GELPs are closely associated with stomata development and are involved in plant response to abiotic stress. A total of 19 putative GELPs control stomatal dynamics, development, and plant water composition in *Arabidopsis* [10]. GELP is required for wax biosynthesis of stomatal cuticular and affected plant drought tolerance in *Arabidopsis* [11]. Soybean GELP28 can enhance the drought and salt tolerance of plants [2], but its biological function is still unknown in vivo.

*Dendrobium* is a large subfamily of orchids including *D. chrysotoxum*, *D. huoshanense*, *D. catenatum*, etc. [12,13]. *D. catenatum* is an important herb in southeast China, which has valuable medicinal components, such as polysaccharides, alkaloids, terpenoids, and flavonoids [14]. Recently, a total of 74 terpene metabolites are identified in *D. catenatum* and a high content of amyrenones is first found in the root [15]. Amyrenones have anti-hyperglycemic, lipid-lowering, and anti-obesity effects *in vivo* [16]. Thus, *D. catenatum* growth and development deserve attention. Our previous transcriptomic analysis hints that photosynthesis and membrane lipids are affected during freezing treatment (FT) and post-recovery freezing (FR) [17]. Proteome and lipidome analyses were further performed to investigate the lipid turnover during freezing and thawing. GELP family members play important roles in plant growth and lipid metabolic regulation [1,18]. Thus, we want to create a supporting basis for the functional prediction of the GELPs family in *D. catenatum* and identify the key candidate GELPs genes for further detailed functional study.

## 2. Results

### 2.1. Comprehensive Identification of GDSL Esterase/Lipase Protein (GELP) Family

**Figure 1:**
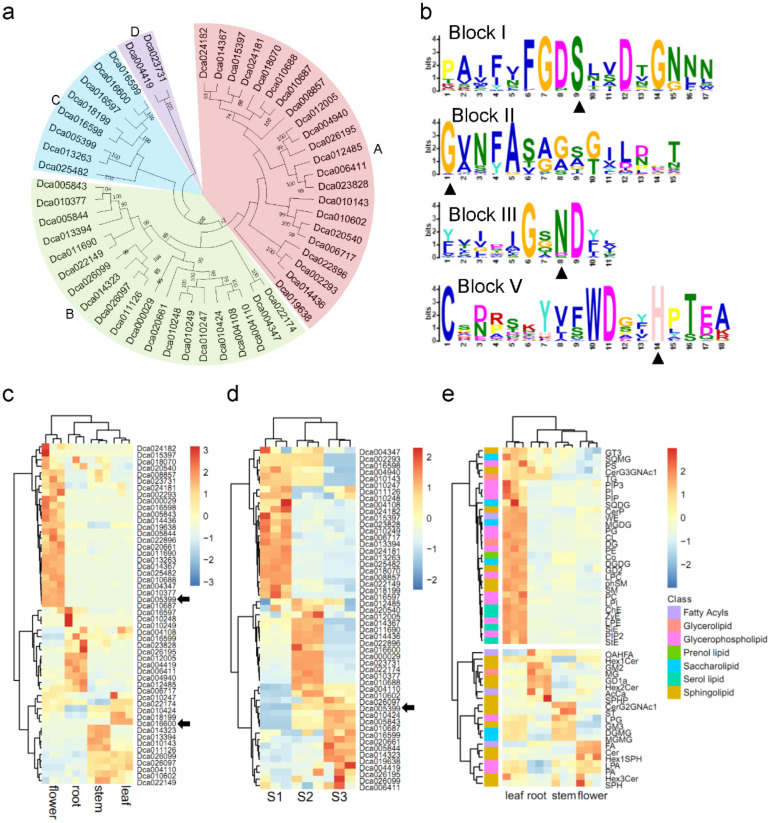
Comprehensive identification of GELP family. (**a**) Phylogenetic analysis of GELPs in *D. catenatum*. A total of 52 GELPs were used to construct the unrooted maximum-likelihood phylogenies. (**b**) Domain organization of GELPs. Conservative blocks (Block I, II, III, and V) of GELPs were shown from up and down. Conservative amino acid residues Ser-Gly-Asn-His in blocks are marked by black triangles. (**c**) Expression patterns of GELP genes in different tissues. (**d**) Expression patterns of GELP genes in three flowering phases. S1, the flower buds were green in the early developmental stage; S2, the flowers had purple pigmentation in the columns and the lips; S3, the sepals and petals had turned yellow and red. (**e**) Lipidome analysis of four tissues in *D. catenatum*. Raw data is shown in Table S1. Lipid abbreviations is listed in Table S2. Color scales represented the values of log_2_ in gene expression levels or lipid content.

Based on the phylogenetic analysis of 52 GELPs from *D. catenatum* genome, the GELP family was divided into four groups (Figure 1a). A total of 20 conserved motifs were predicted with MEME and displayed in Figure S1. Motifs 1, 2, 3, and 4, respectively, represented the conserved blocks I, II, III, and V of the GELP family, which are present in almost all proteins (Figure 1b). To investigate the expression profiles of the *GELP* family in *D. catenatum* growth, two transcriptome data sets were selected for analysis [19,20]. The organ-specific expression patterns indicated that half of *GELPs* were primarily expressed in flowers (Figure 1c). Furthermore, expression levels of *GELPs* were detected during different flowering phases. About half of all GELPs were highly expressed in S1, and the rest were highly expressed in S2 and S3, respectively (Figure 1d). GELPs biological activity was tightly correlated with lipid metabolism. Seven lipid categories were identified from the lipidome (Table S1). Clustering analyses of the lipidome of four tissues revealed that most of the lipids were highly accumulated in leaf tissue. Three sphingolipids and one glycerolipid were highly accumulated in the root. Fatty acid (FA), ceramides (Cer), and hexosyl sphingosine (Hex1SPH) were highly accumulated in flowers (Figure 1e). These results hinted that organ-specific expression patterns of GELPs were associated with lipid tissue distribution.

### 2.2. Identification and Characterization of Dca016600

**Figure 2:**
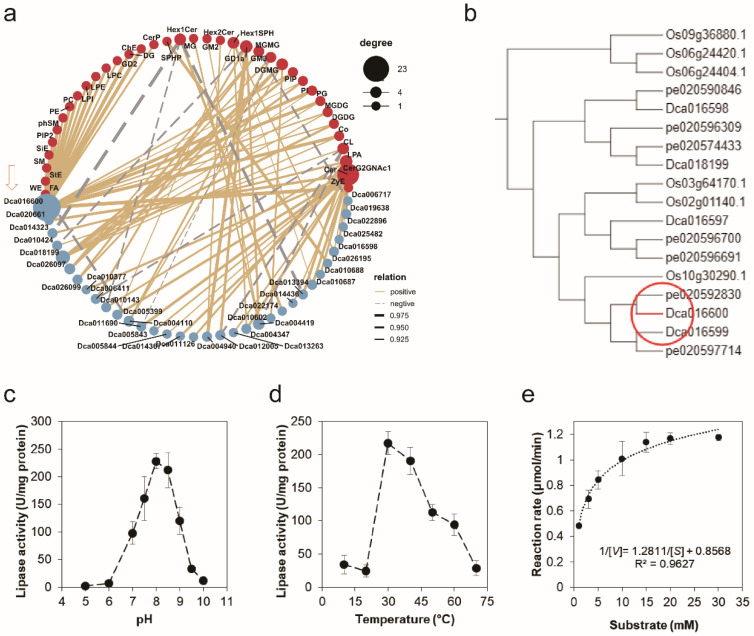
Identification and characterization of Dca016600. (**a**) Correlation analysis of lipidome and GELP expression levels. The dotted and solid lines, respectively, represent positive and negative correlations. The thickness of the line is determined by a Pearson correlation coefficient >0.9 or <−0.9, respectively. The dot sizes and colors represent the correlated number of lipids and genes. (**b**) Phylogenetic analysis of Dca016600 was intercepted to Supplemental Figure S2. Effects of pH (**c**), temperature (**d**), and the effect of substrate concentrations (**e**) for Dca016600 activity. Data represent the mean ± SD of three independent experiments. Km and Vmax values are determined using Lineweaver-Burk plot in Figure S4.

To explore the key *GELPs* involved in lipid metabolism, the co-expression analysis revealed that 38 lipids and 35 *GELPs* were highly correlated (Pearson correlation coefficient > 0.9 or <−0.9), which generated 72 positively correlated pairs and 11 negatively correlated pairs (Figure 2a). Among them, *Dca016600* had 23 positively correlated pairs with lipids (Figure 2a). A total of 504 GELPs from six species of plants were used to construct a phylogenetic tree (Figure S2). This result indicated that Dca016600 was close with *O. sativa* and *P. equestris* homologs (Figure 2b). However, these homologous proteins had no reported function. The recombinant Dca016600-His protein, which had a molecular weight of 33.28 kDa (signal peptide was cut off), was expressed in *E. coli* and was purified for lipase activity assay (Figure S3). Dca016600 was active from pH 7.0 to pH 9.0, with the optimum pH at 8.0 and 8.5 (Figure 2c). The optimum temperature of purified Dca016600 was 30 °C, but it was still active at 40 °C (Figure 2d). Under optimum reaction conditions, 30 °C and pH 8.0, V_max_ and K_m_ of purified Dca016600 were detected and calculated as 233.43 μmol·min^−1^·mg^−1^ and 1.49 mM by Lineweaver-Burk plot, respectively (Figure 2e and Figure S4). These results suggest that Dca016600 may be responsible for intracellular lipid catabolism.

### 2.3. Identification and Expression Analysis of Dca005399

**Figure 3:**
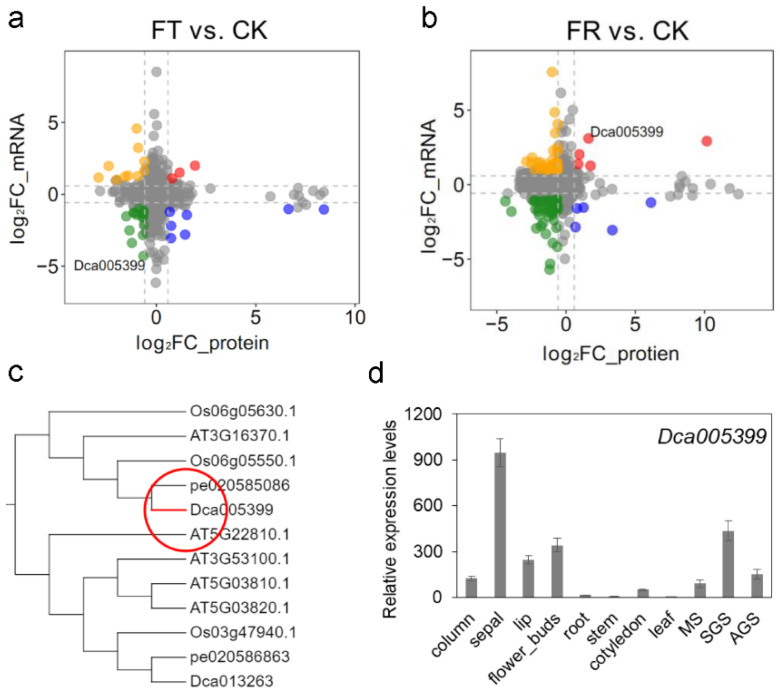
Identification and expression analysis of Dca005399. (**a**) The relationship between changes in protein and mRNA abundances in FT vs. CK. (**b**) The relationship between changes in protein and mRNA abundances in FR vs. CK. The colored points indicate significant upregulation or downregulation of protein and mRNA levels. CK, control condition; FT, freezing treatment; FR, post-freezing recovery. (**c**) Phylogenetic analysis of Dca005399 was intercepted to Supplemental Figure S2. (**d**) Tissues expression analysis of *Dca005399*. Data represent the mean ± SD of three independent experiments. MS, mature seed; SGS, symbiotic germination seed; AGS, asymbiotic germination seed.

*D. catenatum* was greatly affected by cold damage during winter. Our previous study had been performed using metabolome and transcriptome to reveal the response of *D. catenatum* during freezing (FT) and post-freezing recovery (FR) [17]. Furthermore, only one lipase, Dca005399, was significantly changed during FT and FR at the transcriptional and translation levels (Figure 3a,b). The protein and mRNA expression levels of Dca005399 were significantly decreased in FT vs. CK, while significantly increased in FR vs. CK. Phylogenetic analysis showed that Dca005399 was close with AT3G16370 of *Arabidopsis* (Figure 3c). *AT3G16370* (*GGL19*) was preferentially expressed in leaf guard cells, filaments, and sepals [10]. Our data also found that *Dca005399* was highly expressed in flowers, especially in sepals (Figure 1c and Figure 3d). Interestingly, *Dca005399* showed evident expression in seed germination, and especially high expression in symbiotic germination seed (Figure 3d). These results suggested that Dca005399 had important roles in cold stress, plant development and growth.

## 3. Discussion

GELPs have broad substrate specificity and maintain a high number of family members. More than 100 members have been identified in different plant species [2,4,8]. In *Arabidopsis*, the phylogenetic analysis reveals that 105 GELPs are divided into four classes and half of them are expressed in special tissues. For example, the flower-specific expressed genes, *AtGELP42* and *AtGELP83*, improve pollen hydration on the stigma in the early pollination stage [4,21]. A total of 194 GELP genes are identified in the soybean genome and most of them show very low or no transcriptional abundance in plant growth and different tissues. Among them, the overexpression of *GmGELP28* enhances the drought and salt tolerance in plants [2]. However, the number of GELPs in *D. catenatum* and its close specie *P. equestris* are less than half of GELPs in rice, *Arabidopsis*, and *S. moellendorffi* (Figure S2). The endosperm accumulates different types of storage compounds to support the seedling during early post-germinative growth [22]. The hydrolysis of stored lipids in the endosperm by lipase plays a crucial role during seed germination [5]. In all orchid species, the endosperm is absent from the seed, including *D. catenatum* [12]. The lack of endosperm in orchids may therefore be related to the reduction in the GELPs family. Another possible reason may be that *D. catenatum* reference genome is not very well assembled and leads to the deficiency of sequences annotation.

GELPs have been identified in several important economic crops, and several GELPs have been successfully cloned and characterized, primarily in *Arabidopsis*, rice, and tomato [23]. However, there have been no reports on lipases from orchids. We integrate multi-omics data sets and identify two key GELPs in *D. catenatum*. The purified Dca016600 protein shows the optimum temperature is active from 30 °C to 40 °C, and the optimum pH is active from 8.0 to 8.5 (Figure 2). *Dca016600* is primarily expressed in leaves and has a highly positive correlation with 23 lipids (Figure 1c and Figure 2a). These results provide a valuable reference for the study of the Dca016600 function. Another lipase Dca005399 is close to GGL19 of *Arabidopsis* (Figure 3c). *GGL19* is widely expressed in various tissues of each growth phase of *Arabidopsis*, including the early seedling stage, true leaves, and reproductive stage [10]. While *Dca005399* is primarily expressed in mature flowers (Figure 1c,d). In detail, we find that *Dca005399* is highly expressed in sepals (Figure 3d). Consistent with the flowers of other orchids, *D. catenatum* has several distinguishing features in its floral morphology. The columns are derived from the fusion of stamens and pistils. The three petal-like sepals are light green during the early developmental stages of flowering and turn yellow during the full-bloom stage [19]. Thus, *Dca005399* may also be involved in regulating floral organ development or fragrance composition, possibly with redundancy. Moreover, *Dca005399* is involved in seed germination and shows an especially high expression in symbiotic germination seeds (Figure 3d). GELPs participating in the hydrolysis of stored lipids in the initial stage of seed germination have been reported [5]. It is well known that the seeds of almost all orchids rarely germinate in natural conditions. *D. catenatum* seeds depend on mycorrhizal fungi to induce their germination [12]. Thus, we speculate that *Dca005399* mediates the regulation of symbiotic germination and is induced by infection of mycorrhizal fungi.

In recent years, various studies have combined multi-omics data sets to reveal biological progress in plants. Multi-omics technologies, including genome, epigenome, transcriptome, proteome, and metabolome, provide more possibilities to study non-model species. The GELP family contains plenty of members in *D. catenatum*. By routine gene expression analysis, it is hard to find the key candidate genes during stress. With integrated transcriptome, lipidome, and proteome analysis, we screen two GELPs may involve the regulation of different tissues and environments. *Dca016600* is primarily expressed in leaves and has lipase activity in vivo. *Dca005399* is primarily expressed in flowering and is specially induced in symbiotic germination. Although orchid plants lack endosperm, we think that it is important to lipid metabolism in *D. catenatum* seed germination.

## 4. Materials and Methods

### 4.1. Plant Materials

*D. catenatum* (two-year-old) was grown in soil in the greenhouse of Zhejiang University (Hangzhou, China) under conditions of 25 ± 2 °C (12 h light/12 h dark), 80 μmol photons m^−2^s^−1^, and 65–75% relative humidity [14]. Surface-sterilized seeds of *D. catenatum* were germinated on 1% (*w*/*v*) Murashige and Skoog (MS) agar medium for asymbiotic germination. For symbiotic germination testing, surface-sterilized seeds were cultured with *Tulasnella* sp. In oatmeal agar medium (0.25% oatmeal and 1% agar) under 25 ± 2 °C (12 h light/12 h dark). Four tissues and seed samples of *D. catenatum* were immediately frozen in liquid nitrogen for detection. 

### 4.2. Lipidomic Analysis

Lipids were extracted according to a previous study [24]. The lipidomics and data analyses were performed by Shanghai Applied Protein Technology Co., Ltd. (Shanghai, China) [25]. Briefly, samples were grounded into powder in liquid nitrogen and mixed into 440 µL internal standard solution. A volume of 800 µL of methyl tert-butyl ether (MTBE) was incubated with extraction for 30 min at room temperature. After centrifugation, the organic solvent layer was dried under nitrogen. The lipid extracts were re-dissolved in 200 µL 10% ACN/isopropanol and 3 µL of the solution was injected into UHPLC (Nexera LC-30A, Shimadzu, Japan) using CSH C18 column (1.7 µm, 2.1 mm × 100 mm, Waters, Milford, MA, USA). The filtrate was separated by a linear gradient of 30% to 100% ACN/isopropanol (1:9, *v*/*v*) containing 0.1% formic acid and 0.1 mM ammonium formate with a flow rate of 300 μL min^−1^. ESI parameters of Q-Exactive Plus (Thermo Scientific, Waltham, MA, USA) are set as follows: 300 °C source temperature; 350 °C capillary temperature, 3000 V ion spray voltage, 200–1800 *m*/*z* scan range, 50% S-Lens RF level. Lipid species were identified by LipidSearch Software (Thermo Scientific, Waltham, MA, USA) based on 5 ppm mass tolerance of fragment and 5% product ion threshold.

### 4.3. Phylogenetic Analysis

To excavate the homologs of GELPs in the *D. catenatum*, the hidden Markov model (HMM) file of GELP (PF00657) was provided from the PFAM website (http://pfam.xfam.org/; accessed on 1 September 2022). HMMER 3.0 was used to search the GELPs genes from *D. catenatum* reference genome [12] and the cutoff value was set to 0.01. The phylogenetic tree was calculated using the Neighbor-Joining (NJ) method of MEGA X, with the following parameters: Poisson model, pairwise deletion, and 1000 bootstrap replications [26]. The iTOL webpage tool (https://itol.embl.de/; accessed on 12 September 2022) was used to draw the phylogenetic tree [27]. The MEME online program (http://meme.nbcr.net/meme/intro.html; accessed on 12 September 2022) was used to identify the conserved motifs. The SignalP web server (http://www.cbs.dtu.dk/services/SignalP/; accessed on 12 September 2022) was used to analyze signal peptides.

### 4.4. Dca016600 Activity Analysis

*Dca016600* sequence was amplified from *D. catenatum* cDNA using the forward primer 5′- CATATGTCTGGTGGCTGTGGATTTGATCCTC-3′ paired with the reverse primer 5′- AAGCTT ATTTAGTGATGCACCATATTTCTGG-3′. The fragment was ligated into the pET28a vector by *Nde*I and *Hind*III digestion. The construct was transformed into *E. coli* BL21 for Dca016600 protein expression. The protein was purified using Ni-NTA resin (Sangon, China) according to the previous study [24]. Purified protein was used for lipase activity assays according to the methods described previously, one enzyme unit was defined as the amount of enzyme that produced 1 μmol of *p*-nitrophenyl per min [28]. Briefly, 985 μL of substrate solution containing 30 μM *p*-NPP and 50 mM Tris-HCl buffer (pH 8.0) was incubated at 30 °C for 10 min. The substrate solution then was mixed with 10 μL of 0.5 M CaCl_2_ and 5 μL of enzyme solution (contained 1 μg protein) at 30 °C for 10 min. Reactions were stopped by the addition of 200 μL of methanol. UV-visible detection was performed at 405 nm. To assess the effect of pH on the enzyme activity, the substrate solution was chosen 50 mM different buffers (pH 5.0–6.0 citrate, pH 7.0 sodium phosphate, pH 8.0 Tris-HCl, and pH 9.0–10.0 Glycine-NaOH). To assess the kinetic curve of the enzyme activity, the substrate solution contained 1, 3, 5, 10, 15, 20 and 30 mM *p*-NPP, respectively. Reaction conditions were as above.

### 4.5. Real-Time Quantitative PCR

Total RNA was extracted from seed samples using the TransZol reagent (TransGen Biotech, Beijing, China). RNA solution was treated with DNaseI (NEB, Hert, UK) to clear DNA. First-strand cDNA was transcripted from the RNA template by reverse transcription using the TIANscriptRTKit according to the manufacturer’s instructions (TransGen Biotech, Beijing, China). The real-time quantitative PCR processes were performed according to our previous study [29].

### 4.6. Data Analysis

Transcriptome data set of FT and FR were supported by a previous study [17]. Proteome and lipidome data were treated by hierarchical clustering using the R package pheatmap (v1.0.12) and by PCA using the R package FactoMineR (v2.6) according to our previous study [15]. GO enrichment analysis was used in the R package GOplot (v1.0.2) and clusterProfiler (v4.2.2). For DAPs (differential accumulation proteins) selection, protein levels of two comparisons were determined by FC (fold change) > 1.5 or FC < 0.7 and with a statistical significance (*p*-value < 0.05). DALs (differential accumulation lipids) of comparisons were selected by FC > 2 or FC < 0.5, with a statistical significance (*p*-value < 0.05).

## Data Availability

All data generated or analyzed during this study are included in this published article and its Additional files. The datasets generated and analyzed during the current study are available from the corresponding author on reasonable request.

## Supplementary Materials

The following are available online at https://www.mdpi.com/article/10.3390/life12101563/s1, Figure S1: The conserved motifs analysis of GELPs. Four blocks (Block I, II, III and V) were showed in Figure 1a, Figure S2: Phylogenetic analysis of GELPs in *Arabidopsis thaliana* (115 GELPs), *Dendrobium catenatum* (52 GELPs), *Oryza sativa* (122 GELPs), *Phalaenopsis equestris* (61 GELPs), *Selaginella moellendorffi* (145 GELPs), and *Chlamydomonas reinhardtii* (9 GELPs). Total of 504 GELPs were used to construct the unrooted maximum likelihood phylogenies, Figure S3: Dca016600 was expressed in E. coli cells and was purified for the enzymatic activity assay, Figure S4: Lineweaver-Burk plot for Dca016600 activity, Table S1: Lipidomics classification in four tissues, Table S2: Lipid abbreviations list.
