# Supplementary material for: Identification of Two GDSL-Type Esterase/Lipase Genes Related to Tissue-Specific Lipolysis in Dendrobium catenatum by Multi-Omics Analysis
Source: Life (Basel). 2022 Oct 9;12(10):1563. doi: 10.3390/life12101563 (PMC9604673; doi:10.3390/life12101563)
Supplement: Supplementary file 1 [file life-12-01563-s001.zip › Supplementary_Figure.pptx]

## Slide 1
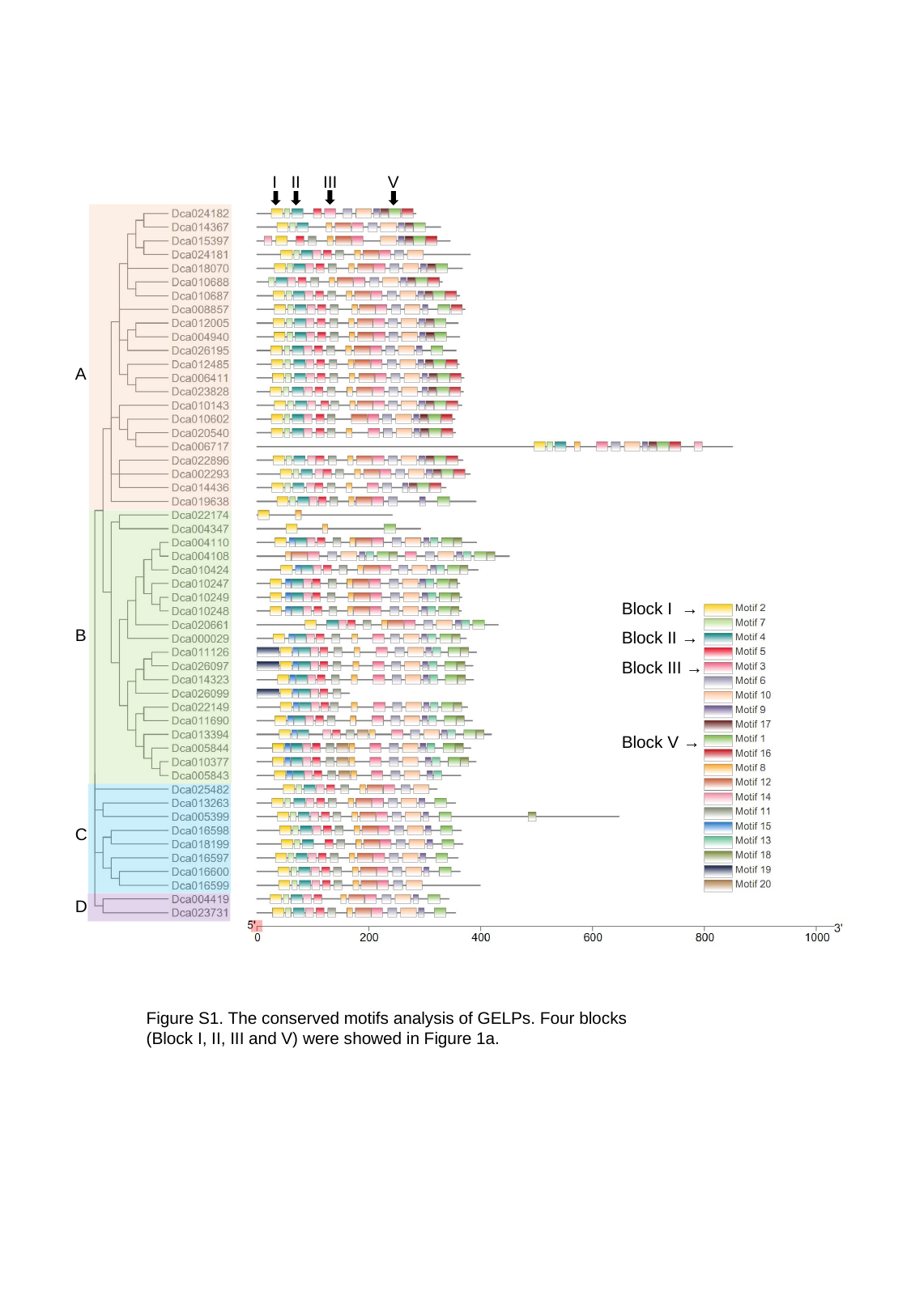

I
II
III
V
Block I →
Block II →
Block III →
Block V →
A
B
C
D
Figure S1. The conserved motifs analysis of GELPs. Four blocks (Block I, II, III and V) were showed in Figure 1a.

## Slide 2
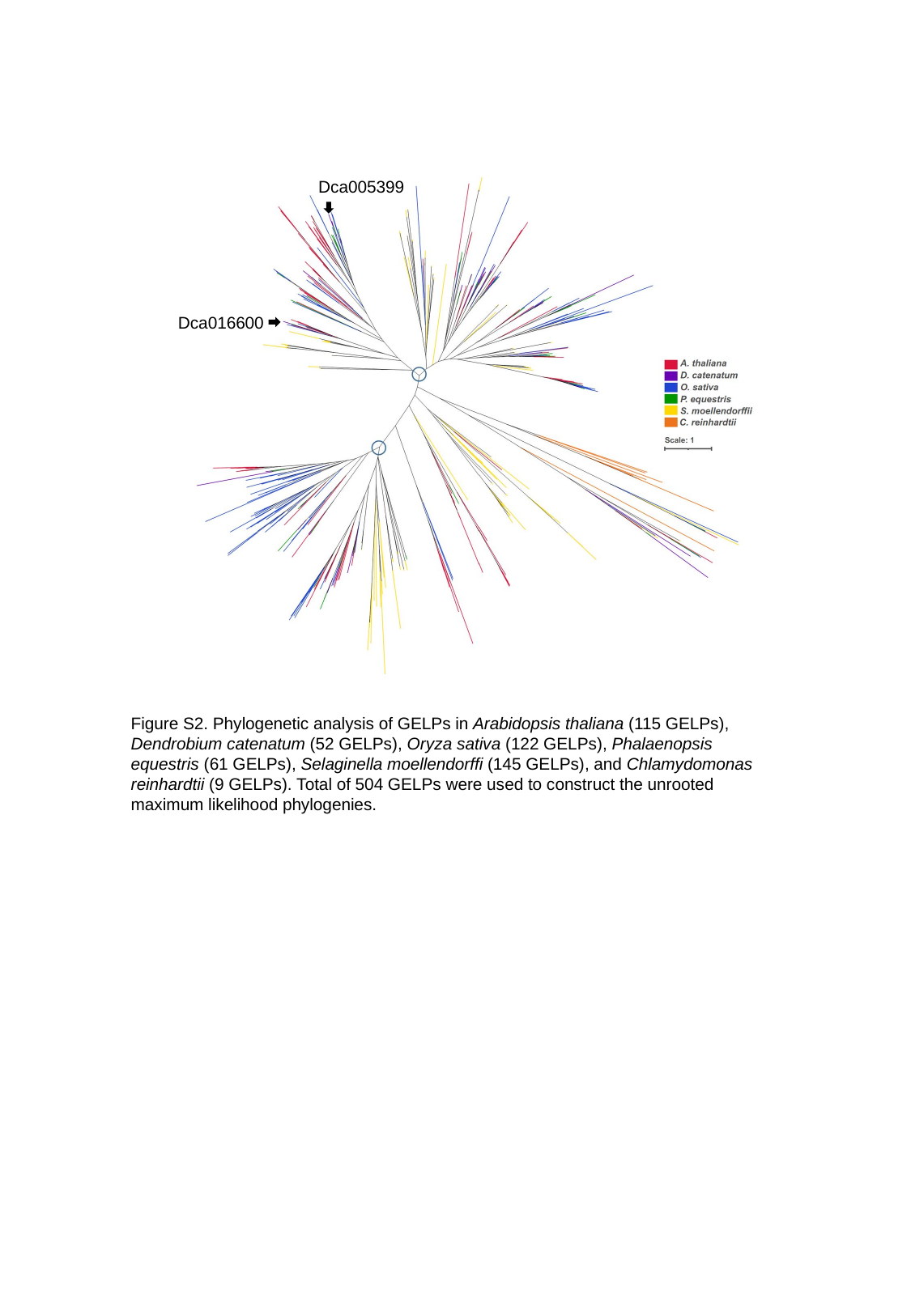

Dca005399
Dca016600
Figure S2. Phylogenetic analysis of GELPs in Arabidopsis thaliana (115 GELPs), Dendrobium catenatum (52 GELPs), Oryza sativa (122 GELPs), Phalaenopsis equestris (61 GELPs), Selaginella moellendorffi (145 GELPs), and Chlamydomonas reinhardtii (9 GELPs). Total of 504 GELPs were used to construct the unrooted maximum likelihood phylogenies.

## Slide 3
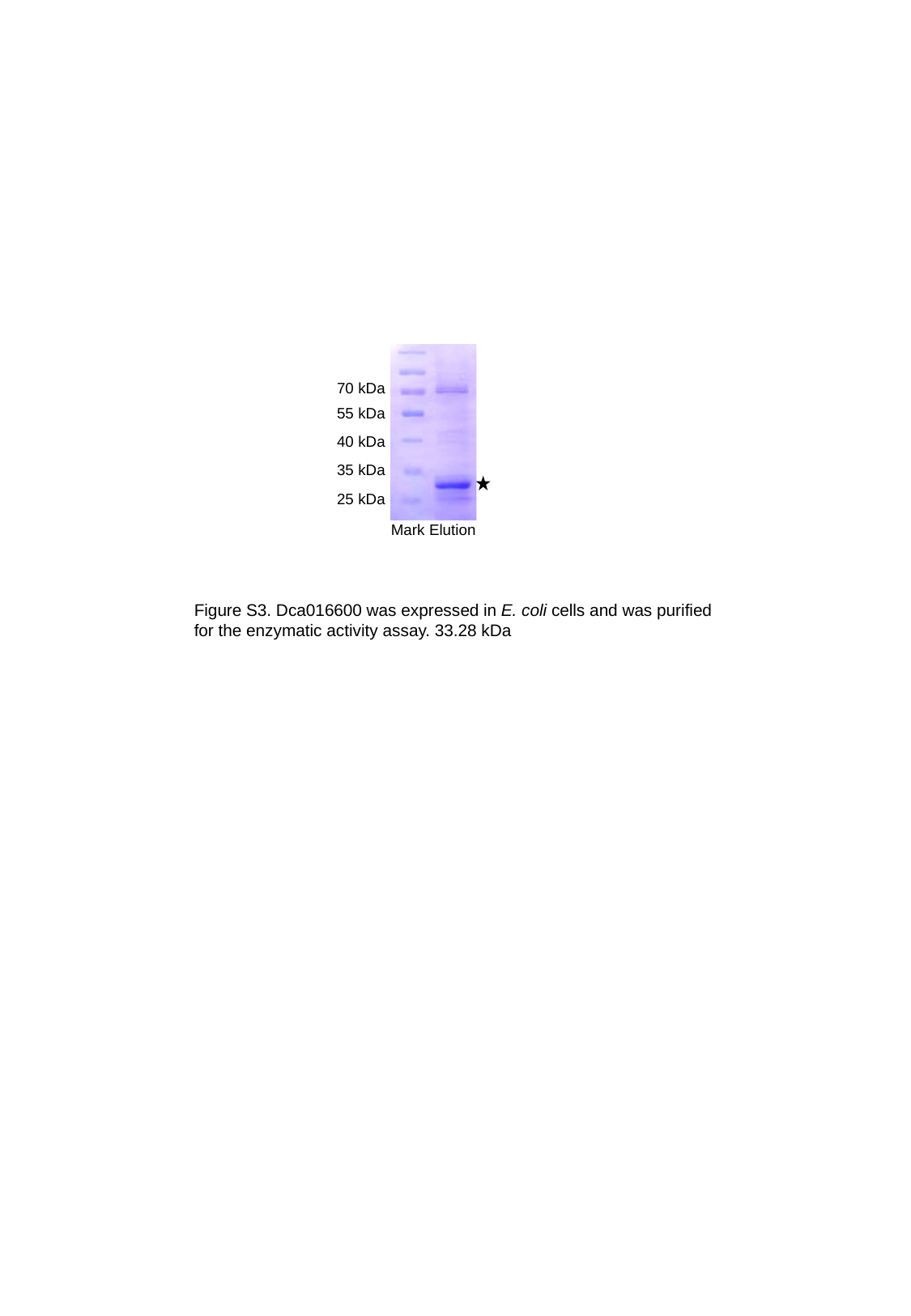

70 kDa
55 kDa
40 kDa
35 kDa
25 kDa
Mark Elution
Figure S3. Dca016600 was expressed in E. coli cells and was purified for the enzymatic activity assay. 33.28 kDa

## Slide 4
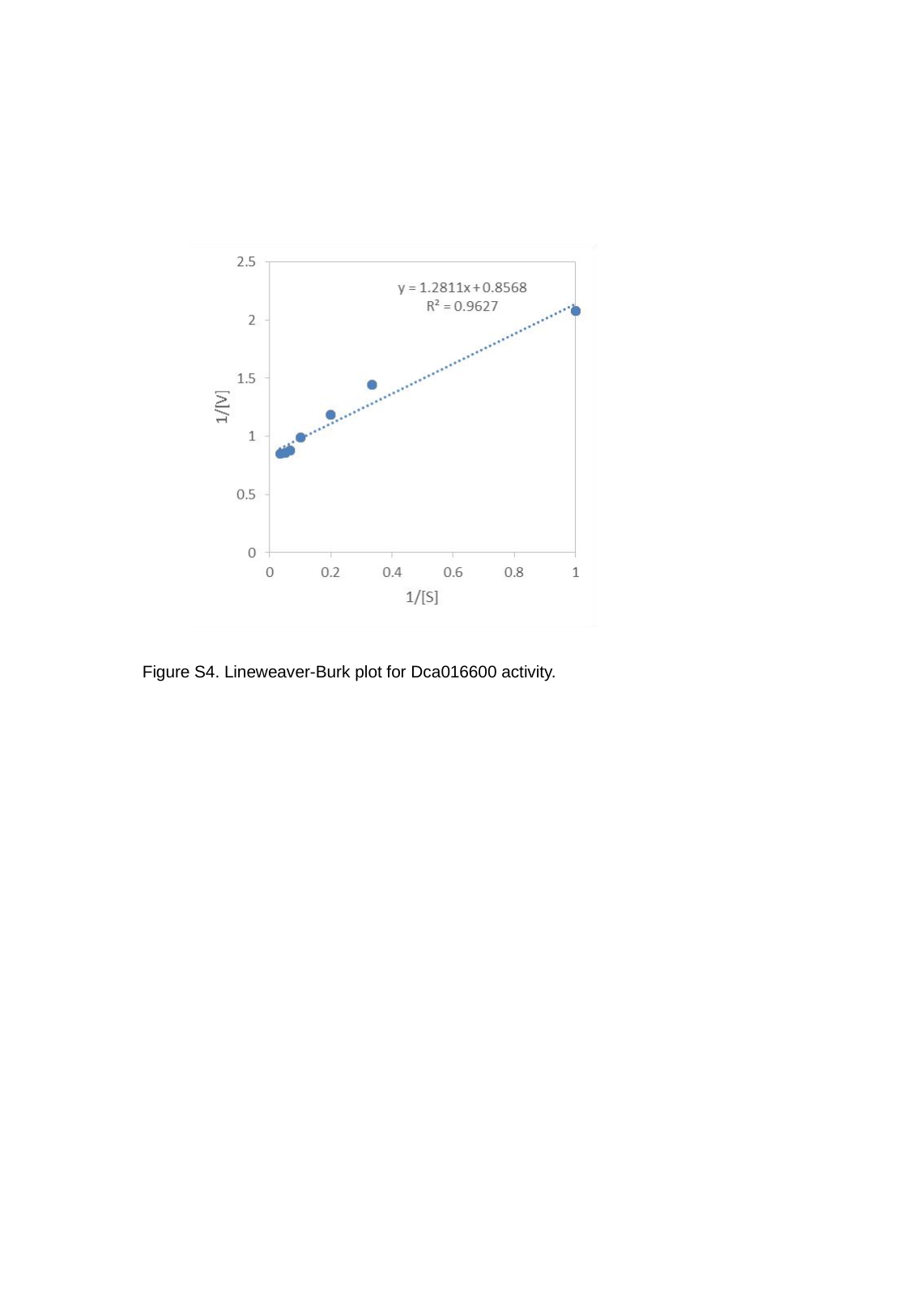

Figure S4. Lineweaver-Burk plot for Dca016600 activity.
